# Oral *Streptococcus mutans* level in high caries risk infants

**DOI:** 10.3389/fdmed.2025.1716923

**Published:** 2026-01-12

**Authors:** Bella Weijia Luo, Ka Fung Yu, Pei Liu, Hai Ming Wong, Edward Chin Man Lo, May Chun Mei Wong, Gillian Hiu Man Lee

**Affiliations:** Faculty of Dentistry, The University of Hong Kong, Pokulam, Hong Kong SAR, China

**Keywords:** dental caries, dental plaque, infant, saliva, *Streptococcus mutans*

## Abstract

**Background:**

The objective of this study was to assess the level of *Streptococcus mutans* in the saliva and plaque of infants with a high caries risk.

**Methods:**

A self-administered parental questionnaire was used to identify one-year-old infants with high caries risk and to collect demographic information, the parents’ and infants’ oral health related behaviors. Saliva and plaque samples were collected from the participating infants. The real-time polymerase chain reaction assays were used to detect the quantity of MS in infants’ saliva and plaque. A clinical examination was conducted to record the caries experience and oral hygiene level of infants.

**Results:**

579 high caries risk infants were recruited. The mean MS count in saliva and plaque was 0.08 ± 0.48 (×10^7^) CFU/mL and 5.72 ± 91.93 (×10^7^) CFU, respectively. Only a few infants had high MS% (>0.03%) in total bacterial count in plaque (4.8%) or saliva (7.3%). The MS level in plaque was found to have a significantly positive association with the prevalence of infants with a white spot or cavitated lesion (*P* = 0.039), but not for the MS level in saliva. Infants from families with 2 or more children (*P* = 0.029) and those who had been fed 7 times or more per day (*P* = 0.020) had a higher likelihood of having a high MS level (MS% > 0.03%) in dental plaque.

**Conclusions:**

MS level in plaque and saliva was generally low among infants. The MS level in plaque showed a clearer association with the onset of dental caries than the salivary MS level in young children.

## Introduction

Early childhood caries (ECC) affects many children worldwide and is a global burden. The disease can begin early in life and progress rapidly when left untreated, especially in those who are at high risk (1). ECC can lead to widespread health issues, affecting children's general well-being and quality of life.

More than 1,000 bacterial species were identified in the oral cavity (2). The bacteria primarily associated with dental caries are *Streptococcus mutans* and Lactobacilli present in plaque (3). *Streptococcus mutans* occurs more frequently around carious lesions (4). Candida albicans is often found together with *Streptococcus mutans* in the plaque of children with ECC (5).

Children acquire cariogenic bacteria from their mothers, other family members, caregivers, or friends through behaviors such as kissing or sharing food or utensils (6). The maternal level of cariogenic bacteria dictates the extent of colonization in their children. The colonization of cariogenic bacteria may occur soon after the eruption of the first primary teeth (7). Earlier colonization of cariogenic bacteria is associated with more severe dental caries among children (8).

Salivary microbiome dynamics have been used to predict the onset of ECC among children aged four to six years (9). Caries activity tests using saliva and plaque have been introduced to detect cariogenic bacteria and identify high caries risk individuals in dental practice. However, the evidence supporting the use of caries activity tests is not strong, particularly for young children. The prevalence of *Streptococcus mutans* in children younger than 2 years old remains unclear. Reports on the levels of *Streptococcus mutans* in the saliva and plaque of infants are limited.

This study aimed to assess the level of *Streptococcus mutans* in the saliva and plaque of one-year-old infants with high caries risk using real-time polymerase chain reaction (real-time PCR). Additionally, it aimed to explore potential associations between bacterial levels and sociodemographic backgrounds of infants and their oral health-related behaviors.

## Materials and methods

### Study population

A self-administered parental questionnaire was used to identify one-year-old infants with high caries risk using the caries risk assessment form for children aged 0–5-year-old endorsed by the American Academy of Pediatric Dentistry (AAPD) (10). An infant was defined as high caries risk when having one of the conditions: (i) Primary caregivers had active dental caries; (ii) Family monthly income was at a median level or below in Hong Kong; (iii) Infants were fed seven times or more per day (more than 3 snacks per day in addition to the 3 regular meals); (iv) Infants had the habit of sleeping with milk bottle. Parent-infant dyads were sequentially recruited between November 2018 and June 2019.

The inclusion criteria were: (i) Children aged 12–16 months; (ii) Children medically fit and healthy and had no antibiotic medication within 3 months before the examination; (iii) At high caries risk; (iv) With at least one tooth erupted; (v) Parents understand Chinese and can complete questionnaires themselves; and (vi) Written consent was obtained. Interested parent-infant dyads fulfilling the inclusion criteria were scheduled for an in-person visit at the Prince Philip Dental Hospital, the dental teaching hospital affiliated with the University of Hong Kong.

### Ethical considerations

The study was reviewed and approved by the Institutional Review Board (IRB) of the Hospital Authority Hong Kong West Cluster (IRB number: #UW17-128). The study was conducted in full accordance with the ethical principles, including the World Medical Association Declaration of Helsinki. Informed consent was obtained from all individual participants.

### Questionnaire

A parental self-completed structured questionnaire was used to collect demographic information, the parents’ oral health related behaviors, feeding habits and oral hygiene practices on their infants (Table 1).

**Table 1:** Sociodemographic background and oral health-related behaviours of the parent-infant dyads.

Primary caregivers	*n* (%)
Relationship with the infant	
Mother	518 (89.5%)
Father	61 (10.5%)
Education level	
Below college	182 (31.4%)
College or above	397 (68.6%)
Monthly household income	
Median or below	149 (25.7%)
Above median	430 (74.3%)
Number of children in the family	
1	466 (80.5%)
2 or above	113 (19.6%)
Had previous oral health education	
Yes	268 (46.3%)
No	311 (53.7%)
Had current oral health problems	
Yes	403 (69.6%)
No	176 (30.4%)
Caregivers’ self-report on own oral health condition	
Good	193 (33.3%)
Fair or poor	386 (66.7%)
Caregivers’ self-report on infants’ oral health condition	
Good	368 (63.4%)
Fair or poor	212 (36.6%)
Infants	*n* (%)
Gender	
Boy	306 (52.8%)
Girl	273 (47.2%)
Had current oral health problems	
Yes	75 (13.0%)
No	504 (87.0%)
Frequency of feeding per day	
6 times or below	208 (35.9%)
7 times or above	371 (64.1%)
Had sweet snacks habit	
Yes	413 (71.3%)
No	166 (28.7%)
Sleeping with milk bottle	
Yes	348 (57.2%)
No	231 (42.8%)
Had mouth cleaning habit	
Yes	526 (90.8%)
No	53 (9.2%)
Had toothbrushing habit	
Yes	342 (59.1%)
No	237 (40.9%)
Had used toothpaste	
Yes	107 (18.5%)
No	472 (81.5%)

(*n* = 579).

### Clinical examination

A trained and calibrated dental professional (BWL) assessed each infant's oral health conditions and collected the saliva and plaque samples. Calibration sessions were conducted regularly in the dental teaching hospital with an experienced pediatric dentist (GL) prior to the study. The dental caries status and oral hygiene level of the infants were recorded.

### Dental caries assessment

The Modified International Caries Detection and Assessment System (ICDAS II, codes 0–6) was used to assess different stages of caries experience, from enamel to dentine (11). White spot lesions (WSL, ICDAS II codes 1 and 2), cavitated lesions (CL, ICDAS II codes 3–6), and the number of teeth with CL (dt) were recorded.

### Oral hygiene assessment

Plaque index (PI) (score 0, 1, 2, 3) was used to record the amount of dental plaque accumulation on every surface of all erupted teeth (12). Mean plaque index per surface was calculated (total plaque score divided by the number of examined surfaces of all erupted teeth). The prevalence of infants with visible plaque (VP) was indicated by codes 2 and 3.

### Collection of saliva and plaque samples

Parents were instructed not to feed their infants at least an hour before the dental examination. At the end of the dental examination, supragingival dental plaque samples from the infants were collected by gently rubbing the buccal surfaces of all erupted incisors with a sterile cotton bud, which were then immediately immersed in a sterile bottle containing 1.0 mL of sterile phosphate-buffered saline (pH 7.0). After plaque sampling, unstimulated saliva (≥100 μl) was collected with a sterile plastic pipette into 5 mL tubes and kept on ice (<4 °C) during transport. The plaque was washed from the cotton bud into 1.0 mL PBS and centrifuged at 16,000 × g for 3 min, repeated three times. The pellet was then stored at −70 °C. Saliva samples were stored at −70 °C within 6 h of collection.

### Quantification of *Streptococcus mutans*

All the saliva and plaque samples from the infants underwent DNA extraction and PCR amplification to evaluate the composition of oral *Streptococcus mutans* (MS) in the participating infants.

All the bacterial DNA in the whole saliva (100 μl saliva for each infant) and plaque samples were extracted following the modified protocol of the DNA extraction kit (Gentra® Puregene® Extraction Kit, Qiagen Cat. No:158567, Gentra Puregene Handbook, 2014). The extracted bacterial DNA from the saliva and plaque samples was then frozen at −70 °C for real-time polymerase chain reaction (PCR) analysis.

Real-time PCR assays of *Streptococcus mutans* were performed with StepOne® Real-Time PCR System. PCR amplification of the prepared DNA samples was conducted by quantitative real-time polymerase chain reaction (Q-RT-PCR) using “Species-specific” SYBR green One-Step Q-RT-PCR Kit (Applied biosystems® Power SYBR® Green PCR master mix, Thermo Fisher Scientific, REF:4367659). A standard curve was generated using DNA extracted from a pure MS strain (UA159).

Reactions were carried out prior to PCR amplification. The reaction mixture (20 μl) per well in the plate contained 10 μl SYBR® Green PCR master mix, 7 μl diluted water (H_2_O), 1 μl forward primer (MS: gtfB-forward; universal bacteria: u16s-forward), 1 μl reverse primer (MS: gtfB-reverse; universal bacteria: u16s-reverse), and 1 μl standard DNA sample. Considering both methodological consistency and biological relevance, gtfB was chosen as the sole qPCR target, as supported by the literature (13–16). The target sequence of the MS primer was designed according to the instructions provided by Applied Biosystems® Power SYBR® Green PCR master mix (REF:4367659). The novel universal primer was used in this study (17). The details of the primers are described in Box 1.

Box 1 **Details of the primers.**

**Table:** 

gtfB- forward	5’-CGCACCACACGGACTTCA-3’
gtfB- reverse	5’-TGGTCAAGAGTAAAGGTCGGTAAG-3’
u16s- forward	5’-ACTCCTACGGGAGGCAGCAGT-3’
u16s- reverse	5’-TATTACCGCGGCTGCTGGC-3’

The 96-well plate containing the reaction mixture was set in duplicate and placed into the StepOnePlus@ Real-Time PCR Systems for analysis. The machine ran the process automatically and took around two hours to analyze one plate. The entire analysis process included initial denaturation for 10 min at 95 °C, followed by 40 cycles of denaturation for 15 s at 95 °C, and primer annealing for 1 min at 60 °C. Melting curve analysis was conducted for each sample to verify specificity.

The MS count and total bacterial count were measured by quantitative PCR amplification performed in duplicate. The ratio of MS count in the total bacteria count (the sum of bacterial cells in the saliva or plaque sample) was then calculated.

### Data analysis

Data analysis was conducted using IBM SPSS Statistics 30 (SPSS Inc., Chicago, IL, USA). All the data were treated with strict confidentiality. Characteristics of the study participants were presented with descriptive statistics. Chi-square tests were used to compare the proportions of MS levels between groups. Multiple logistic regression analysis was used to assess the associations between sociodemographic background and infants’ oral health-related behaviors (as listed in Table 1) and the MS level (MS% > 0.03% vs. MS% ≤ 0.03%). Insignificant variables were removed using backward stepwise selection. The level of statistical significance for all tests was set at *P* < 0.05.

## Results

A total of 579 parent-infant dyads participated in the study. Their sociodemographic background and oral health-related behaviors are presented in Table 1.

The primary caregivers were predominantly mothers of the infant (89.5%). The mean age of caregivers was 34.0 ± 4.3 years (range: 21–50 years). Less than half (46.3%) of them had oral health education before. One-third (33.3%) of caregivers rated their own oral health as good, and nearly two-thirds (63.4%) thought their infants were in good oral health condition.

There were more boys (52.8%) than girls (47.2%). The mean age of the infants was 14.3±  1.0 months (range: 12–16 months). The majority of the infants (80.5%) were the only children of the family. More than half of infants (64.1%) were fed seven times or more per day. Over half of caregivers (59.1%) performed toothbrushing for their infants, but only 18.5% used toothpaste. The average number of teeth present was 8.4 ± 2.9.

### Oral health condition of infants

Table 2 presents the oral health condition of the infants. The WSL prevalence was 10.71%. The CL prevalence among infants was 1.04%, with a mean dt score of 0.03 ± 0.30; none of the lesions were restored. The prevalence of infants with WSL or CL was 11.05%.

**Table 2:** Oral health conditions of the infants.

Dental caries experience	% (*n*)
Infants with cavitated lesion	1.04% (6/579)
Infants with white spot lesion	10.71% (62/579)
Infants with white spot lesion or cavitated lesion	11.05% (64/579)
dt (mean ± SD)	0.03 ± 0.30
Oral hygiene level	
Infants with visible plaque	40.07% (232/579)
Plaque index (mean ± SD)	0.40 ± 0.41

(*n* = 579).

The prevalence of infants with VP was 40.07% (232/579) with a mean plaque index (PI) of 0.40 ± 0.41 per surface of all erupted teeth. More than one-third of infants had visible plaque on at least one tooth surface; however, the average plaque level was actually low.

### *Streptococcus mutans* (MS) level

#### In plaque

The MS could not be detected in one-third (33.2%) of the infants, and only a few (4.8%) of them had a high MS level with MS% > 0.03% (percentage of MS count in the total bacterial count). The mean MS% was 0.13% ± 1.73% with a maximum of 38.12%. The majority of infants had a very low level of MS in the plaque. The MS count in each plaque sample was detected. The mean MS count in plaque was 5.72 ± 91.93 (×10^7^) CFU (Colony-Forming Unit), and the median count was 0.01 × 10^7^ with a maximum count of 2,167.42 × 10^7^. Infants with MS% > 0.03% had a mean MS count of 116.28 ± 409.36 (×10^7^) CFU, while infants with MS% ≤ 0.03% had a mean MS count of 0.10 ± 0.78 (×10^7^) CFU (Table 3).

**Table 3:** *Streptococcus mutans* (MS) level in the plaque and saliva samples of infants.

		Per plaque sample	Per saliva sample
MS%		(*n*, %)	(*n*, %)
Not detected		192, 33.2%	243, 42.0%
<0.001%		218, 37.7%	81, 14.0%
0.001%–0.01%		115,19.9%	152, 26.3%
0.01%–0.03%		26, 4.5%	61, 10.5%
>0.03%		28, 4.8%	42, 7.3%
Mean ± SD		0.13%±1.73%	0.03%±0.42%
Median		0.0002%	0.0005%
Minimum		0	0
Maximum		38.12%	9.86%
Percentiles	25	0	0
	50	0.0002%	0.0005%
	75	0.0015%	0.0056%
MS count (CFU×10^7^)		Per plaque sample	Per 1 mL saliva sample
Mean ± SD		5.72 ± 91.93	0.08 ± 0.48
Median		0.01	0.02
Minimum		0	0
Maximum		2167.42	8.77
Percentiles	25	0	0
	50	0.01	0.02
	75	0.09	0.05

CFU, colony-forming unit; MS%, MS count in the total bacterial count×100%. (*n* = 579).

#### In saliva

The MS could not be detected in more than two-fifths (42.0%) of infants, and only a few (7.3%) had a high MS level with MS% > 0.03%. The mean MS% was 0.03%±0.42% with a maximum of 9.86%. The majority of infants also had a very low level of MS in their saliva. The MS count per 1 mL saliva sample was detected and calculated. The mean MS count in per 1 mL saliva sample was 0.08 ± 0.48 (×10^7^) CFU, and the median count was 0.02 × 10^7^ with a maximum count of 8.77 × 10^7^. Infants with MS% > 0.03% had a mean MS count of 0.05 ± 0.16 (×10^7^) CFU, while infants with MS% ≤ 0.03% had a mean MS count of 0.005 ± 0.02 (×10^7^) CFU (Table 3).

### Association between MS level and infants’ caries experience

The MS level in plaque was found to have a significantly positive association with infants’ caries experience (*P* = 0.039). Infants who had a high MS level (MS% > 0.03%) in plaque had a higher proportion of having a WSL or CL (Table 4).

**Table 4:** Association of oral *Streptococcus mutans* level with infants’ caries experience.

	Infants with WSL or CL	
	Presence (%)	Absence (%)
MS% in plaque		
Not detected	8.9	91.1
<0.001%	11.5	88.5
0.001%–0.01%	10.4	89.6
0.01%–0.03%	7.7	92.3
>0.03%	28.6	71.4
*P*-value^#^	0.039*	
MS% in saliva		
Not detected	11.1	88.9
<0.001%	11.1	88.9
0.001%–0.01%	9.9	90.1
0.01%–0.03%	16.4	83.6
>0.03%	7.1	92.9
*P*-value^#^	0.620	

CL, cavitated lesion; WSL, white spot lesion; MS%, MS count in the total bacterial count×100%. (*n* = 579).

# Chi-square test.

* *P* < 0.05, statistically significant.

In contrast, regarding the MS level in the saliva, no statistically significant associations were found between MS level and the infants’ caries experience (*P* = 0.620).

### Association between MS level, sociodemographic background and infants’ oral health-related behaviors

MS level (high: >0.03% vs. low: ≤0.03%) in saliva did not reveal any significant associations with sociodemographic background factors and infants’ oral health-related behaviors (those listed in Table 1) (Table 5).

**Table 5:** Association of high MS level with sociodemographic background and infant's oral health-related behaviors.

Factor	MS%>0.03% in plaque^a^	Chi-square test	Multiple logistic analysis	
		*P*-value	*OR (95%CI)*	*P*-value
Number of children in the family				
1^b^	3.9%	0.027*		0.029*
≥2	8.8%		2.456 (1.094, 5.513)	
Infants’ frequency of feeding per day				
≤6 times^b^	1.9%	0.014*		0.020*
≥7 times	6.5%		3.571 (1.218, 10.467)	
Infants sleeping with milk bottle				
No^b^	2.6%	0.041*		
Yes	6.3%			

MS%, MS count in the total bacterial count×100%. OR, odds ratio.

a Proportion of infants with MS%>0.03% in the plaque sample.

b Reference category.

* *P* < 0.05, statistically significant.

MS level in plaque was found to have significant positive associations with the number of children in the family (*P* = 0.027), infant's feeding frequency per day (*P* = 0.014) and infants’ habit of sleeping with a milk bottle (*P* = 0.041) using the Chi-square test.

Multiple logistic regression analysis, using backward stepwise selection, identified two independent variables in the final model. Infants from families with 2 or more children (*P* = 0.029) and those who had been fed 7 times or more per day (*P* = 0.020) had a higher likelihood of having a high MS level (MS% > 0.03%) in dental plaque.

## Discussion

This was the first study with a large sample size (>500) to report the *Streptococcus mutans* (MS) level in infants’ mouths with high caries risk. Our findings showed that MS level in plaque was positively associated with caries experience of the infants, whereas no such association was found with the MS level in saliva.

The real-time PCR technique (18) was used extensively to detect the presence of bacteria and viruses. In this study, PCR assays with the gftB primer were used to quantify MS in infants’ saliva and plaque samples (13–20). The percentage of MS count in the total microbial count (MS%) in each sample was used to quantify the MS level. Since it was challenging to collect the same amount of saliva and plaque samples from each participating infant, the outcome (MS%) made the comparison feasible.

The strength of this study was the large sample size used to assess MS levels in plaque and saliva in such young infants, as the prevalence of MS in children younger than 2 years remains unclear, and reports are limited. On the other hand, the “window of infectivity” of MS in infants is still controversial. Despite the challenges of collecting samples from such young infants, this effort was valuable and made a significant contribution to this field of research, addressing the research gap.

Regarding the limitations, one major challenge was collecting saliva from a one-year-old infant, which is particularly difficult given their uncooperative behavior. Sometimes, it's difficult to obtain saliva from their mouth, even with their parents’ help, and the amount of saliva collected may not be consistent in quality or quantity. Thus, to ensure consistency, only 100 μl from each saliva sample was used for DNA extraction, ensuring a fair comparison. The sampling procedures were standardized to minimize potential sources of bias. The parents were asked not to feed their infants for at least an hour before the dental examination, and the same collection steps were performed in the clinical setting. DNA extraction and PCR were performed in the laboratory according to the appropriate protocol. Another limitation was that only MS was used as the indicator of dental caries risk, even though dental caries is a multifactorial disease that involves other pathogens, and numerous microbial factors contribute to caries pathogenesis. Nevertheless, MS is the major contributor to bacterial involvement. Collecting mothers’ oral health status would enable further exploration of the transmission of MS from mothers to their children. However, the data analyzed in this study were collected from a randomized clinical trial at baseline, which focused on the caries-preventive effectiveness of two types of toothpaste, so the mothers’ oral health conditions were not available.

The association of MS with the prevalence of dental caries has been established (10, 21, 22). Previously, the initial acquisition of MS was assumed to occur during the “window of infectivi’ when children are aged 19–31 months (23). However, more recent studies found that MS colonization could occur at an earlier age, even as early as 8–15 months (24, 25).

Although the mean age of our participating infants was relatively young (14.3 ± 1.0 months), compared with other studies, a higher proportion of infants in our study had MS colonization in both plaque and saliva. Two-thirds of them (67%) already had MS in plaque compared to 27% in 1-year-old infants (26) and 41% in 18-month-old infants (27). Over half of the infants (58%) had MS in saliva, compared to 33% (27) and 30% (28) in 18-month-old infants. This is because all participating infants had a high caries risk, as assessed using the caries risk assessment form for 0–5-year-olds endorsed by the AAPD (10). Therefore, their likelihood of acquiring MS in their daily lives was higher.

In our study, a high MS level was defined as MS% > 0.03%, as there was a significant difference in the mean MS count compared with MS% ≤ 0.03%. Infants with this high MS level in plaque had a significantly higher likelihood of having a cavitated lesion or white spot lesion, and were also associated with more than one child in the family, infants having 7 times or more feedings per day and the habit of sleeping with a milk bottle. All these factors could promote the MS colonization in the mouth. Therefore, MS% > 0.03% in plaque may represent a potential threshold for indicating risk in infants, which could be explored further in future studies.

The number of children in the family was significantly associated with a high MS level (MS% > 0.03%) in plaque. Infants who were the family's only child had lower odds of having a high MS level in plaque. Big families have a higher likelihood of sharing food or utensils, and hence, it is easier to acquire MS from family members through vertical or horizontal transmission (6). Furthermore, the parents may have more time to take care of their only child's oral hygiene, reducing the opportunity for MS colonization.

Infants had higher MS levels in plaque when they had poor oral health-related behaviors, including sleeping with a milk bottle and a frequent feeding habit (fed 7 times or above per day). Children sleeping with a milk bottle were found to have a higher caries risk and promote MS colonization (10, 29, 30). Parents should prepare to wean their baby off the bottle and slowly transition to a cup at 1 year old (31). The biofilm (dental plaque) started to form on the tooth surfaces after feeding. Therefore, infants fed seven times or more per day had higher MS levels.

However, there was no association between MS level in saliva and the infants’ caries experience, and it also did not have any significant associations with various sociodemographic factors, oral health conditions and practices. Over two-fifths of the infants (42%) did not have any MS detected in saliva. The diversity of the infant salivary microbiome increased as they aged from 3 to 24 months old (32). The prevalence and abundance of selected oral microbes changed with age. Salivary MS level is low in young children. Using salivary MS level of infants or young children to predict the onset of ECC or to determine their caries risk may not be as sensitive or practical. Infants, especially at this young age, were unable to expectorate saliva into specimen bottles. It was challenging to gain cooperation and collect their saliva.

The early colonization of MS among the participating infants indicated the need for early oral health care. The World Health Organization (31) recommended the first dental visit by age one year, and AAPD (33) suggested establishing a dental home for children before the child's first birthday. Our findings support these guidelines, suggesting that preventive counseling and tailored oral hygiene measures should be delivered during the early dental visit to reduce the risk of early MS colonization and subsequent caries development.

The families recruited in this study were invited to participate in a randomized controlled trial (RCT), a two-year longitudinal prospective clinical study comparing the effectiveness of caries prevention between two different toothpastes (fluoride toothpaste VS xylitol toothpaste) in high-caries-risk children. The RCT results will be reported separately.

## Conclusions

The MS level in plaque and saliva was generally low in one-year-old infants with high caries risk. The MS level in plaque showed a clearer association with the onset of dental caries than the salivary MS level in young children. Still, the acquisition of MS can occur at an early age, particularly for infants with high caries risk. Preventive dental care should be delivered early to control MS colonization.

## Data Availability

The raw data supporting the conclusions of this article will be made available by the authors, without undue reservation.
